# Metabolomic Reconfiguration in Primed Barley (*Hordeum vulgare*) Plants in Response to *Pyrenophora teres* f. *teres* Infection

**DOI:** 10.3390/metabo13090997

**Published:** 2023-09-07

**Authors:** Claude Y. Hamany Djande, Fidele Tugizimana, Paul A. Steenkamp, Lizelle A. Piater, Ian A. Dubery

**Affiliations:** Research Centre for Plant Metabolomics, Department of Biochemistry, University of Johannesburg, P.O. Box 524, Auckland Park, Johannesburg 2006, South Africa; claudeh@uj.ac.za (C.Y.H.D.); ftugizimana@uj.ac.za (F.T.); psteenkamp@uj.ac.za (P.A.S.); lpiater@uj.ac.za (L.A.P.)

**Keywords:** barley, *Pyrenophora teres*, dichloroanthranilic acid, hydroxycinnamic acids, metabolomics, net-blotch disease, priming, defence-related metabolites

## Abstract

Necrotrophic fungi affect a wide range of plants and cause significant crop losses. For the activation of multi-layered innate immune defences, plants can be primed or pre-conditioned to rapidly and more efficiently counteract this pathogen. Untargeted and targeted metabolomics analyses were applied to elucidate the biochemical processes involved in the response of 3,5-dichloroanthranilic acid (3,5-DCAA) primed barley plants to *Pyrenophora teres* f. *teres* (*Ptt*). A susceptible barley cultivar (‘Hessekwa’) at the third leaf growth stage was treated with 3,5-DCAA 24 h prior to infection using a *Ptt* conidia suspension. The infection was monitored over 2, 4, and 6 days post-inoculation. For untargeted studies, ultra-high performance liquid chromatography coupled with high-resolution mass spectrometry (UHPLC–MS) was used to analyse methanolic plant extracts. Acquired data were processed to generate the data matrices utilised in chemometric modelling and multi-dimensional data mining. For targeted studies, selected metabolites from the amino acids, phenolic acids, and alkaloids classes were quantified using multiple reaction monitoring (MRM) mass spectrometry. 3,5-DCAA was effective as a priming agent in delaying the onset and intensity of symptoms but could not prevent the progression of the disease. Unsupervised learning methods revealed clear differences between the sample extracts from the control plants and the infected plants. Both orthogonal projection to latent structure-discriminant analysis (OPLS-DA) and ‘shared and unique structures’ (SUS) plots allowed for the extraction of potential markers of the primed and naïve plant responses to *Ptt*. These include classes of organic acids, fatty acids, amino acids, phenolic acids, and derivatives and flavonoids. Among these, 5-oxo-proline and citric acid were notable as priming response-related metabolites. Metabolites from the tricarboxylic acid pathway were only discriminant in the primed plant infected with *Ptt*. Furthermore, the quantification of targeted metabolites revealed that hydroxycinnamic acids were significantly more prominent in the primed infected plants, especially at 2 d.p.i. Our research advances efforts to better understand regulated and reprogrammed metabolic responses that constitute defence priming in barley against *Ptt*.

## 1. Introduction

Understanding plant defence mechanisms is critical in plant breeding practices to control plant disease. The rising global food demand and the indiscriminate application of chemical fertilisers highlight the necessity for sustainable crop production systems. Novel approaches for improved resistance are required to address these concerns, as phytopathogenic microorganisms represent a severe threat to global food production and ecosystem stability. Plants use an intricate and multi-layered innate immune system to ward off attacks by potential pathogens present in the environment. This involves the activation and regulation of signalling cascades and actions at the gene expression-level and protein biosynthesis-level, as well as the structural reinforcement of the cell wall [1,2,3]. 

Barley (*Hordeum vulgare* L.) is one of the most versatile cereals cultivated in different regions. However, the crop has shown susceptibility to fungal pathogens, including the ascomycete *Pyrenophora teres* f. *teres* (*Ptt*), the causal agent of the ‘net blotch net form’ (NBNF) disease. Worldwide, net blotch is among the most prevalent destructive disease of barley, leading to enormous yield losses of up to 70% in some cases, depending on the susceptibility of the cultivars, the trends in the environmental conditions, and the virulence of the pathogen [4,5,6,7,8]. This interdependence between the plant’s susceptibility, the pathogen’s virulence (both having strong genetic links), and environmental factors is illustrated in the disease triangle (Figure S1). The triangle is complete when all of the following three conditions are met: a susceptible host, a virulent pathogen, and a favourable environment. An alteration or manipulation of one triangle component may affect the disease’s progression [9,10]. 

Plant–pathogen interactions commence immediately after the host makes contact with the microbe-associated molecular pattern (MAMP) molecules derived from the pathogen. The latter uses a wide spectrum of physical and/or enzymatic tools to access plant nutrients. In turn, the plant recognises the pathogen signals and activates constitutive pre-formed and specific inducible defence systems. Fungal infections significantly impact the physiology of the host, including its gene expression profiles, primary metabolism, oxidative stress, and regulation of signalling pathways. Through the phenomenon known as priming, plants can be prepared or induced to easily adjust to such environmental changes, resulting in a faster and more efficient response to the pathogen and helping the plant to resist the attempted attack, thus enabling it to sustain less damage.

Despite lacking an adaptive immune system, it is now well known that plants can ‘remember’ or’ learn’ from previous attacks (e.g., rewiring the stress memory), resulting in stronger and faster responses to low-stimulus concentrations. This phenomenon is known as defence priming, which is regarded as a component of induced resistance (IR) [11]. Through ‘priming’ or ‘preconditioning’ mechanisms, information from the complex immune system may be stored as a ‘memory’, enabling the plant to quickly and more efficiently activate/stimulate defences to restrict pathogen growth and multiplication [11,12]. The primed condition can be established by preconditioning or pretreatment with particular substances, biological agents, or physical factors to increase survivability and the effectiveness of defence mechanisms under potential stress [13,14]. While the mechanism behind defence priming is still unclear, the different phases involved remain, and they are as follows: (i) the priming phase corresponding to the perception of the stimulus; (ii) the post-challenged primed state involving exposure to a secondary stimulus; and (iii) the transgenerational primed state where primed parents pass down the primed state to future generations [12,15,16,17]. 

Metabolites not only define and determine the fundamental physiological and agronomic features of plants but also shape the biochemical dynamics of defence responses to stress conditions, which carries imprints of environmental and genetic factors [18]. In an earlier study [19], we found that treatment with 3,5-dichloroanthranilic acid (3,5-DCAA) as a priming agent orchestrated metabolic perturbations in the investigated barley cultivars. Changes were characterised by the up- and down-regulation of defence-related metabolites and precursors, which define a ‘state of alertness’ in the plants. Mass spectrometry-based metabolomics approaches, targeted or untargeted, are essential components of system biology investigations. Over the last decade, a lot of attention has been paid to applying metabolomics tools and approaches to shed more light on the mechanisms involved in plant–pathogen interactions [20,21,22]. For the present study, the ‘Hessekwa’ cultivar, which has shown susceptibility to *Ptt* in trial studies (Figure S2), was selected to evaluate the effect of the pathogen on naïve and 3,5-DCAA-primed plants. Both untargeted and targeted metabolomics approaches with computational tools were used to monitor and explore the metabolic reconfiguration that occurs in barley plants (primed or naïve) and the characteristics of their response to pathogenic infection. 

## 2. Materials and Methods 

### 2.1. Barley Plant Material and Growth Conditions

‘Hessekwa’ is an experimental barley cultivar that was developed for the Western Cape region of South Africa (a winter rainfall area). The South African Barley Breeding Institute (SABBI, Bredasdorp, Western Cape, South Africa) provided the cultivar seeds. The seeds were grown as previously described [23]. Briefly, the cultivar was grown in a plant growth room under well-controlled conditions, which were as follows: 12 h fluorescent light (≈85 µmol m^−2^ s^−2^) and 12 h dark cycle at 22–27 °C. The seeds were surfaced-sterilised (70% ethanol) and soaked in sterile water for 2 h before they were planted in pasteurised (at 70 °C) soil (Germination mix, Culterra, Muldersdrift, South Africa). About 40 seeds were planted per pot (12 cm in diameter and 8 cm depth). The plants were watered twice a week with distilled water containing a water-soluble chemical fertiliser (Multisol ‘N’, Culterra, Muldersdrift, South Africa). This cultivar was selected based on a preliminary study of five barley cultivars: ‘Erica’, ‘Agulhas’, ‘Elim’, ‘S16’, and ‘Hessekwa’. The results of the preliminary study identified ‘Hessekwa’ as the most susceptible to *Ptt*. In addition to being an important cultivar under development in South Africa, the susceptibility of the cultivar to the pathogen of interest was a part of the criteria for selection. 

### 2.2. Fungal Isolate, Culture, and Sporulation

The *P. teres* f. *teres* isolate W1-1, the causative pathogen of net blotch net form disease (NBNF) on barley, was provided by Dr. Jordi Muria-Gonzalez, Curtin University, WA, Australia. Growth of the fungus first commenced on V8-PDA (V8 vegetable extract-potato dextrose agar) medium and sub-cultured into a barley–oat–agar (BOA, pH 7) solid media for improved sporulation (adapted from [7,24]). The plates were incubated for 10 d under a 12 h/12 h photoperiod at 22 °C. *Ptt* sporulation was induced by placing plates hydrated with 500 µL sterile water under black light (365 nm UV light) for 20 h. To induce conidia formation, fungal plates were further incubated for 24 h at 15 °C in the dark (JM Gonzalez, personal communication). Figure S3 shows a plate before and after enhancing sporulation. For spore collection, 5 mL of sterile water containing 0.05% of Tween-20 was added to the plates, and a soft brush was used to dislocate them. This process was performed twice to maximise the number of conidia collected. The resulting liquid was vortexed and filtered through muslin cloth before determining the conidia concentration using a haemocytometer and light microscope at 40X magnification. 

### 2.3. Plant Treatment/Inoculation and Experimental Design

Barley shoot tissues were treated with 3,5-DCAA 24 h prior to infection (when the plants were 20 d post-planting). The choice to use 3,5-DCAA as a priming agent was based on metabolomic data [19]. The inducer was dissolved in dimethylsulphoxide (DMSO; 1 µL mL^−1^) and subsequently diluted into a buffered wetting agent dissolved in dH_2_O, pH 6.0 (‘Insure’, Efekto, Pretoria, South Africa), to a concentration of 200 µM and sprayed on shoot tissue for systemic uptake. 

The harvested *Ptt* conidia were diluted to a concentration of 1 × 10^5^ mL^−1^, and the suspension was sprayed onto the shoot tissues of the primed and naïve barley plants (5 mL per pot). The plants were incubated for 24 h at 18 °C and in humid conditions to favour conidia solution uptake before returning to the normal above-mentioned growing conditions. The plants were inoculated when they turned 21 d old and were harvested at 2, 4, and 6 d post-infection (d.p.i.). 

Overall, the experimental design included the following: (i) the naïve plants (control; no treatment); (ii) the Tween-20 control, which served as the vehicle control for the *Ptt* conidia; (iii) the DMSO control, which served as the vehicle control for the 3,5-DCAA agent; (iv) 3,5-DCAA, which served as a control for the primed and infected conditions (PI); (v) infected naïve plants (*Ptt*) and (vi) infected primed plants (DCAA + *Ptt*). All of the above were involved in three independent biological replicates. Upon harvesting, the leaves were cut from the roots and snap-frozen in liquid nitrogen to quench metabolic activity. 

### 2.4. Metabolite Extraction and Sample Preparation 

Harvested leaf tissues were pulverised in a mortar and pestle with liquid nitrogen. To 1 g of leaf powder, 10 mL of 80% methanol was added, and the mixture was homogenised using an Ultra-Turrax homogeniser (CAT, Ballrechten-Dottingen, Germany) and a probe sonicator (Bandelin, Sonopolus, Berlin, Germany) set at 55% power for 10 s. The homogenates were centrifuged for 20 min at 5100× *g* and 4 °C, and the resulting hydro-methanolic supernatants were concentrated to 1 mL on a rotary evaporator, transferred to 2 mL Eppendorf tubes, and evaporated further to complete dryness in a dry bath at 45 °C within a fume hood. The extracts were dissolved in 50% methanol and then filtered through 0.22 µm nylon filters into chromatography vials before ultra-high performance liquid chromatography–quadrupole time-of-flight mass spectrometry (UHPLC–qTOF–MS).

### 2.5. Ultra-High Performance Liquid Chromatography–High Definition Mass Spectrometry (UHPLC-HDMS) for Untargeted Metabolomics

The UHPLC–qTOF–MS analyses of the extracts were performed on a Waters Acquity UHPLC hyphenated with a Waters SYNAPT G1 high resolution using accurate mass spectrometer system in V-optics (Waters Corporation, Milford, MA, USA) as a detector. The aqueous methanol extracts were separated using the Waters HSS T3 C18 column (150 mm × 2.1 mm × 1.8 µm) at 60 °C. The T3 column has the advantage of separating compounds ranging from polar to non-polar despite its status as a C18-based reverse phase column. A binary solvent system made up of water (eluent A) and acetonitrile (Romil Pure Chemistry, Cambridge, UK; eluent B) (both containing 0.1% formic acid) was used for the concave gradient elution at a flow rate of 0.4 mL min^−1^. The elution was initiated with 5% B for 1 min and gradually increased to 95% B after over 24 min. The concentration of B was kept constant at 95% for 2 min and finally changed back to the initial conditions after 27 min. Before the next injection, the conditions were set in a manner that allowed the analytical column to calibrate for 3 min. The total run time was 30 min, and each sample’s injection volume was 2 µL. Each sample was examined in three technical replicates to account for analytical variability. Quality control samples (QCs) were run at the beginning and end of the batch and in between (every 15 injections) to monitor the condition of the LC-MS system and evaluate the dependability and reproducibility of the analysis. The sample order was randomised, and blanks (50% methanol) were injected to monitor background noise, the possible carry-over of samples, and any solvent contamination. 

High-resolution, accurate mass MS analyses were operated in negative and positive electrospray ionisation (ESI) modes. The capillary voltage was set at 2.5 kV; the sampling and extraction cone voltages were 40 V and 4.0 V, respectively. The source temperature was fixed at 120 °C, and the desolvation temperature was set at 450 °C. The cone and desolvation gas flows were 50 L h^−1^ and 550 L h^−1^, respectively. Nitrogen was used as the nebulisation gas at a flow rate of 700 L h^−1^. A mass range of 50 to 1200 *m*/*z* was selected with a scan time of 0.1 s. The reference mass calibrant, leucine enkephalin (50 pg mL^−1^, [M–H]^−^ = 554.2615 and [M + H]^+^ = 556.2766) was sampled every 15 s and produced an average intensity of 350 counts per scan. The reference allowed the processing software (MassLynx XS^TM^ 4.1, Waters Corporation, Milford, MA, USA) to automatically correct slight deviations in the centroid masses observed in the samples, thereby providing exact mass measurements. This resulted in a typical mass accuracy of 1 to 3 mDa. In addition, a data-independent acquisition (DIA) method (MS^E^) involving fragmentation at different collision energies (0–40 eV) was applied to generate fragmentation data to comprehensively extract the structural information of detected analytes and assist in metabolite annotation.

### 2.6. LC-ESI-QqQ-MS System for Targeted Metabolite Analyses

The triple quadrupole (QqQ) mass spectrometry platform used was an LC–MS 8050 instrument (Shimadzu, Kyoto, Japan), and it was equipped with an electrospray ionisation (ESI) source and an ultra-fast liquid chromatograph (UFLC) as a front-end. The multiple reaction monitoring (MRM) method was used for the absolute quantification of the targeted metabolites. The MRM-MS conditions were developed and optimised via direct infusion (using ESI source of MS), and the collision energy (CE) was optimised for each transition using the ‘MRM optimisation method tool’, an integral component of LabSolutions LC–MS software (Shimadzu Corporation, Kyoto, Japan). The tool automates the process by collecting product ion scan data and finding the optimum CE for each transition. 

The targeted defence-related compounds were the amino acids phenylalanine (Phe), tyrosine (Tyr), and tryptophan (Trp); phenolic acids such as cinnamic acid, caffeic acid, ferulic acid, and sinapic acid; and, finally, two alkaloids, namely, hordenine and gramine. All the standards were obtained from Sigma-Aldrich (St. Louis, MO, USA) and BDH (Poole, UK). All working solutions of the mixed standards (concentrations ranging from 5 × 10^−2^ µg mL^−1^ to 4.5 µg mL^−1^) were dissolved in 50% MeOH. Both the samples and the working solutions were analysed on the UFLC system using a C18 reverse phase chromatography column (Pinnacle DB Aqueous C18, 100 mm × 2.1 mm, 3 µm particle size) (Restek, Bellefonte, PA, USA). The injection volume was 1 µL, and the constant flow rate was 0.4 mL min^−1^. A binary gradient made of MilliQ water with 0.1% formic acid (eluent A) and pure-grade MeOH (Romil Chemistry, Cambridge, UK) with 0.1% formic acid (eluent B) was used. The elution was initiated with 5% B for 1 min and gradually increased to 95% B after over 25 min. The concentration of B was kept constant at 95% for 2 min and finally changed back to the initial conditions after 27 min. 

The MRM-MS method was developed and used for quantitative MS analyses. The MS conditions were as follows: the heating gas flow was set at 10 L min^−1^, the interface temperature was set at 300 °C, the interface voltage was set at 4 kV, the DL and heat block temperatures were set at 250 °C and 400 °C, respectively. Nitrogen gas was used as a drying gas at a flow rate of 10 L min^−1^ and as a nebulising gas at a flow rate of 3 L min^−1^. The optimal parameters for each metabolite and the equation generated from the standards are reported in Table S1. The concentrations are reported in Table S2.

### 2.7. Data Processing and Data Mining

Data pre-processing was carried out using the MarkerLynx-XS^TM^ application manager of the MassLynx-XS^TM^ 4.1 software (Waters, Manchester, UK). The software creates a matrix with individual mass spectra information, variable retention time (Rt) and *m*/*z* pairs, and integrated and normalised peak areas. The following parameters were used: Rt = 0.7–24 min (ESI^–^) and Rt = 0.7–20 min (ESI^+^) range and 100–1200 Da for the mass range. The mass tolerance was set to vary by up to 0.05 Da, and the Rts was set to vary by up to 0.2 min. The intensity threshold was set at 50 counts. Only data matrices with noise levels under the MarkerLynx cut-off of 50% were considered for further chemometric and statistical studies. The generated data matrices were imported into SIMCA (Soft Independent Modelling of Class Analogy) software, version 14 (Sartorius, Umeå, Sweden), for chemometric analyses. The principal components analysis (PCA) unsupervised approach and a supervised learning model, orthogonal projection to latent structures-discriminant analysis (OPLS-DA), were used within the SIMCA package. The models were consistently validated using permutation (*n* = 100) tests analyses and a seven-fold cross-validation (CV) method computed within the software [21,25]. OPLS-DA-generated loadings ‘S-plots’ were assessed for the selection of variables. Features with both high correlation and covariation were considered, [p(corr) ≥ 0.5, ≤−0.5 and (p1) ≥ 0.1, ≤−0.1], and variable importance in projection (VIP) plots were used to evaluate the statistical significance of each feature (cut-off > 1) and avoid possible bias from occurring during feature selection [16]. In addition, ‘Shared-and-Unique-Structures’ (SUS) plots were generated to reveal the shared and distinct metabolic features of the primed and naïve infected plants. The SUS plots were generated using the p(corr) value obtained in the OPLS-DA models, and they show metabolites that co-ordinately contribute to the primed and naïve infected conditions. Only components contributing to the strength of the prediction capacity of the model (R1 significant components) were considered. 

### 2.8. Metabolite Annotation and Metabolic Pathway and Network Analyses

Metabolite annotation was based on MS data (accurate mass and mass fragmentation patterns) as described [23,26], focusing mostly on discriminant metabolites. The annotations were made according to the Metabolomics Standard Initiative (MSI) level 2 [27]. The estimated molecular formula of a chosen ion (feature corresponding to a metabolite) was manually searched using bioinformatics tools and databases such as PubChem [28], ChemSpider [29], Lipidmaps [30], and the Dictionary of Natural Products [31]. The mass spectral fragmentation patterns were examined and confirmed via comparison with information on barley in the literature and metabolome data from related plants. The MetPA (Metabolomics Pathway Analysis) [32]) module of the MetaboAnalyst bioinformatics platform (Version 5.0) [33] was used to perform pathway analysis on the successfully annotated metabolites that were selected based on the OPLS-DA data. Metabolites with known KEGG (Kyoto Encyclopedia of Genes and Genomics, [34]) identities (KEGG identifiers) were entered into the MetPA tool, and enrichment analysis was used to evaluate the potential biological roles thereof. The pathway analysis calculated the significance of the impacted pathways based on the pathway impact values (*x*-axis) obtained from our pathway topology analysis and the *p* values acquired from our pathway enrichment analysis (*y*-axis). Furthermore, using MetaMapp [35], correlation network analysis was performed for a global visualisation of the metabolic alterations. This was performed utilising the fold-changes and *p* values determined from OPLS-DA-derived descriptive statistics as well as the MetaMapp-encoded chemical structures of the annotated metabolites from the PubChem and KEGG databases (Table S3). The subsequent metabolic networks were displayed using the network visualisation tool Cytoscape (version 3.9.1; [36]), and the similarity cut-off between metabolites was determined using a Tanimoto score threshold of 0.7.

## 3. Results and Discussion

The ability of a plant to overcome a pathogen attack determines the level of resistance thereof. The molecular communication between both organisms depends on the genetic make-up of both participants and may result in resistance by the host or invasion by the pathogen. A resistant (R) plant will successfully overcome the attack, while the pathogen will defeat a susceptible (S) one, causing infectious disease. Partially resistant or tolerant cultivars lie within an R–S continuum, with the outcome frequently determined by abiotic environmental factors. As previously mentioned, resistance can be enhanced, and an IR phenotype can involve both primed and induced defence responses and can be brought into play at a local or systemic level against microbial pathogens that can exhibit varied lifestyles. With xenobiotic agents/inducers like 3,5-DCAA, the relative significance of primed vs. induced defences may be dependent on a range of factors, such as the concentration and chemical nature of the stimulus and the bio-availability and perception thereof, as well as the kinetics of the response and timing of analysis. Moreover, the effectiveness of IR responses can depend on the plant species, the developmental age of the plant, the tissue type, and the virulence of the pathogen [14]. 

### 3.1. Evaluation of the Net Blotch Net Form Disease on Barley

**Figure 1:**
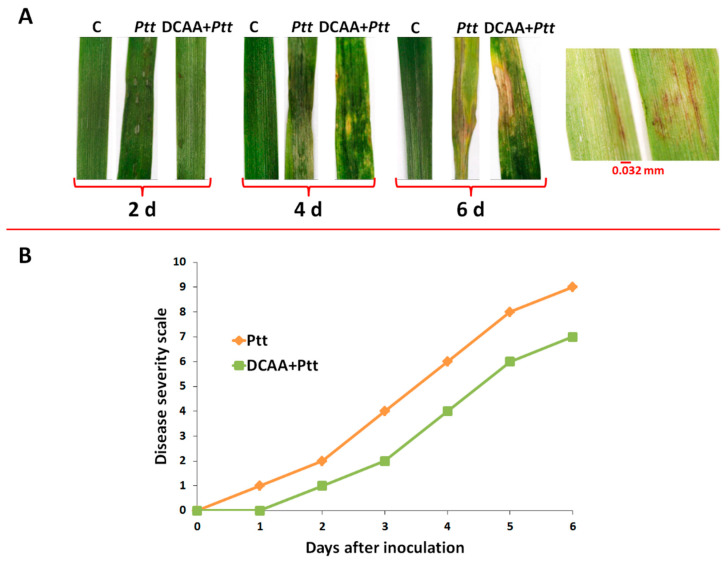
Fungal infection symptoms and disease severity assessment. (**A**): Barley shoot tissue at 2, 4, and 6 d post-inoculation with *P. teres* f. *teres* conidia. (**B**): Net blotch net form (NBNF) disease symptom evaluation on a numerical scale ranging from 0 to 10. 0 = No symptoms; 1 = Resistant; 2 = Resistant to moderately resistant; 3 = Moderately resistant; 4 = Moderately resistant to moderately susceptible; 5 = Moderately resistant to moderately susceptible; 6 = Moderately resistant to moderately susceptible; 7 = Moderately susceptible; 8 = Moderately susceptible to susceptible; 9 = Susceptible; 10 = Very susceptible.

As indicated in the experimental section, the ‘Hessekwa’ cultivar of barley (naïve and pre-treated with 3,5-DCAA) was infected with a 1 × 10^5^ mL^−1^ conidia suspension of the fungal pathogen *Ptt*. This was followed by assessing the plants’ phenotypic characteristics and evaluating the disease severity index. Symptom development and the corresponding disease severity score ranging from 0 to 10 (as reported by Tekauz) [37] are illustrated in Figure 1A,B, respectively. In Figure 1A, an early manifestation of fungal infection (at 2 d.p.i.) was phenotypically characterised by fusiform and circular dot-like lesions, progressing into dark brown longitudinal and transverse striations constituting the net-like pattern on the shoot tissues of the naïve infected plants. From 4 d.p.i., the symptoms on primed and naïve plants are slightly comparable; however, there is a shrinking observed on naïve-treated plants but not on plants pre-treated with 3,5-DCAA. In addition, chlorotic areas could also be highlighted around the necrotic lesions observed on both challenged leaves from 4 d.p.i for the 3,5-DCAA-treated plants and at 6 d.p.i for naïve plants. The zoomed-in photograph highlights the observed net-type lesions (characteristic of the NBNF disease). 

The disease severity index was as described in the legend of Figure 1A. Looking at the disease severity scale shown in Figure 1B, symptoms could be observed as early as 24 h post-inoculation in the naïve infected plants showing a disease index of 1. On the 3,5-DCAA treated shoot tissues, the first symptoms were observed at 2 d.p.i. In both cases, the steady development of phenotypic symptoms was observed to reach a score of 9 (for the naïve plants) and 7 (for the primed plants) at over 6 d.p.i, corresponding to the plant’s susceptibility state. As mentioned in the introduction, disease progression depends on the virulence of the pathogen, the environment, and the susceptibility of the cultivar. Here, environmental variables were controlled, and preliminary studies revealed ‘Hessekwa’ cultivar as susceptible to the *Ptt* W1-1 isolate (Figure S2). 

The NBNF disease is a destructive disease of barley that causes important yield losses in susceptible cultivars. *Ptt* can colonise the host by producing necrotic and chlorotic symptoms due to net blotch in plant tissue [5,38,39]. In susceptible barley cultivars, germinated conidia produce the appressorium through the plant membrane at 1 d.p.i. [40,41]. This is followed by fungal growth inside the leaf tissue, resulting in necrosis and chlorosis from 4 d.p.i. The interaction between the susceptible barley plant and the *Ptt* pathogen results in the colonisation of the plant tissue, the release of nutrients and energy production for fungal growth, the suppression of plant defence responses, and, eventually, induced cell death. The delayed development of symptoms in the 3,5-DCAA-treated plants inversely correlates to the time deployed to respond to the fungal attack. This behaviour supports the post-challenged primed state characterised by the quicker and stronger defence response of the primed plants compared to the naïve infected plants [17,42]. Although the disease severity index of the primed infected plants at 6 d.p.i. is still reduced compared to that of the naïve infected plants, the pathogen can seemingly overcome the host defences at that time point. Subsequently, untargeted and targeted metabolomic approaches were used to pinpoint discriminatory metabolites and identify the biological pathways characterising the responses of the primed and naïve plants to the fungal infections.

### 3.2. Chromatographic and Chemometric Profiling of Primed and Naïve Barley Plants Following Infection by the Fungus P. teres f. teres

**Figure 2:**
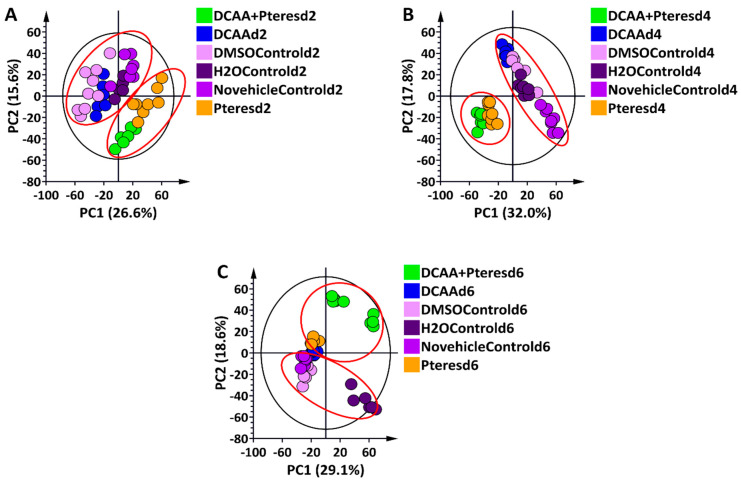
Principal component analysis (PCA) score plots of the ESI (–) data from shoot extracts of the ’Hessekwa’ cultivar of *Hordeum vulgare* treated with 3,5-DCAA and infected with *P. teres* f. *teres*. All data were *Pareto*-scaled, and the calculated Hotelling’s T^2^ with a 95% confidence interval is represented by the ellipses present in each PCA score plot. (**A**): Six-component model of all conditions, d2, explaining 69.2% variation and predicting 45.1% variation; (**B**): Six-component model of all conditions, d4, explaining 74.8% variation and predicting 54.7% variation; (**C**): Seven-component model of all conditions, d6, explaining 72.5% variation and predicting 55.2% variation. The groupings of the controls and the treatments are highlighted in red.

To elucidate the metabolic profiles of the primed and naïve barely plants responding to *P. teres* f. *teres* infection, the methanolic extracts were analysed via high-definition, accurate mass UHPLC-MS. Although the base peak intensity (BPI) chromatograms show only the most intense peaks at a specific retention time, they revealed subtle quantitative and qualitative differences across extracts (Figure S4). The data obtained from our LC-MS analyses were further analysed using multivariate statistical tools to simplify the visualisation of the complex multidimensional datasets, detect outliers, uncover the underlying trends, and extract relevant information for biological interpretation. From the unsupervised method, the computed PCA scores plots (Figure 2A–C and Figure S5), distinct clustering separating all of the control and primed samples from the infected ones was observed at all time-points. Although this result was not so evident on day 2 and day 4, with the ESI (–) data, at day 6, a clear separation of the naïve infected and primed infected clusters was observed. In addition, all control samples (no vehicle control, DMSO control, and Tween-20/H_2_O control), as well as the 3,5-DCAA-treated samples, grouped together on the PCA score plots, showing no major variations between these conditions. As a result, the naïve plant condition (no vehicle control, C) was then used as a comparative reference for primed infected and naïve infected conditions in downstream data mining processes. 

**Figure 3:**
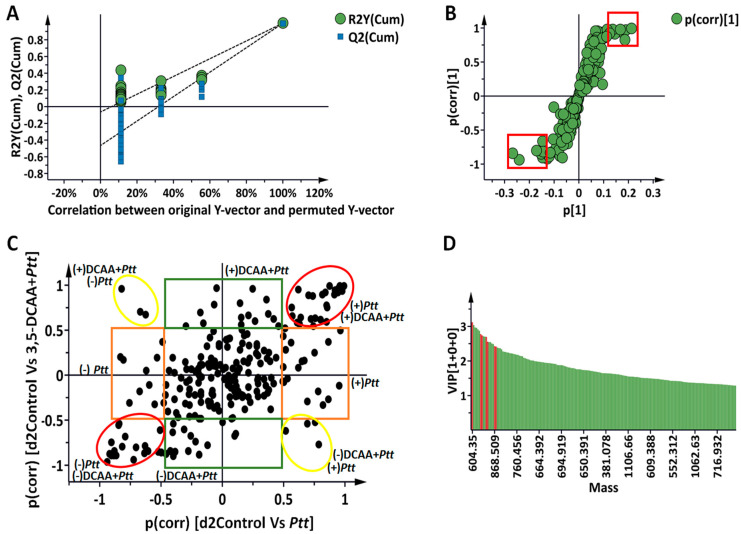
Orthogonal projection to latent structures discriminant analysis: selection of discriminant metabolites at 2 d.p.i. (**A**): Permutation test (n = 100) for the OPLS-DA model of ‘Control vs. *Ptt*’ (x-axis, component 1 + 2 + 0; R^2^X = 0.694, R^2^Y = 0.999, Q^2^ = 0.985. CV-ANOVA = 2.15 × 10^−9^); (**B**): OPLS-DA loading S-plot. The variables highlighted in the red squares are statistically significant in discriminating the two compared groups. The covariance (magnitude) and correlation (reliability) of the samples in the model are represented on the axes as p[1] and p(corr), respectively. (**C**): Shared-and-unique structures (SUS) plot analysis for correlating the predictive component of the OPLS-DA models ’Control vs. *Ptt’* and ’control vs. DCAA + *Ptt’* (y-axis, Component 1 + 2 + 0; R^2^X = 0.789, R^2^Y = 0.994, Q^2^ = 0.975; CV-ANOVA = 3.44 × 10^−8^) at 2 d.p.i. The most discriminant features in both models are highlighted with circles. Yellow circles correspond to the discriminant features inversely correlated between the two models, and red circles indicate those similarly correlating. The unique discriminant features for each model have been highlighted using orange (for *Ptt*) and green (for DCAA + *Ptt*) rectangles. As mentioned, only features with a high correlation were selected (p(corr) ≥ 0.5, ≤−0.5). Up-regulation and down-regulation are shown as positive and negative values, respectively. (**D**): Variable importance in projection (VIP) scores for each selected metabolite >1.

As mentioned in the Materials and Methods section, OPLS-DA was applied as a supervised learning method to characterise and distinguish the classes and groups observed in the profile generated using the above-mentioned explorative models. The evaluation of the OPLS-DA models was performed by conducting a cross-validation (CV)-ANOVA (with a *p* value < 0.05 being considered a significant model) [25]. In addition, out of the hundred randomly permutated models, none outperformed the original ones. Figure 3 (based on data from the 2 d.p.i timepoint) serves as a representative example of the steps followed, with Figure 3A showing the correlation between the original and the permutated Y-vectors, as well as the R^2^ and Q^2^ of the models. The represented permutation analysis plot displays the R^2^ and Q^2^ values of the permutated models (left-bottom corner), which are lower than those of the original (right-top corner). Statistically significant features were selected using the loading S-plots generated from the OPLS-DA data and models (Figure 3B). The S-plots identified features originating from metabolites with high covariation and high correlation that discriminates between the conditions being compared. Furthermore, taking into consideration the aim of this study, which was to investigate the changes that occur after both the naïve and primed ‘Hessekwa’ barley cultivars become infected, another variable selection method, known as the ‘Shared-and-Unique Structures’ (SUS) loadings plot, was used (Figure 3C), and underlying response markers associated with the post-challenged trends observed after employing the unsupervised models could thus be extracted. Only metabolites with high covariance and high correlation and those with a VIP score > 1 were considered potential biomarkers (Figure 3D). All annotated discriminant metabolites or potential biomarkers are summarised in Table S4. 

### 3.3. Defence-Related Metabolic Reprogramming in 3,5-DCAA-Primed and Naïve Barley Plants Following Infection by the Fungus P. teres f. teres

**Figure 4:**
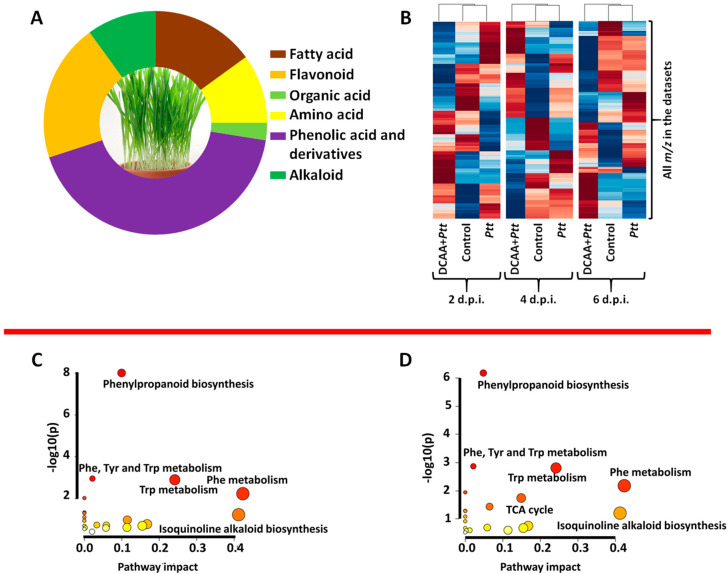
Discriminant metabolites in the 3,5-DCAA-primed and naïve barley plants after infection with *Ptt* and summary of the MetPA-generated metabolic pathways in the naïve and primed infected plants. (**A**): Metabolite class distribution. (**B**): Dendrogram heatmap showing the extent of metabolomic reconfiguration as reflected by the relative concentration of all metabolites across all conditions (Control, DCAA + *Ptt*, and *Ptt*) and all timepoints (2, 4, and 6 d.p.i.). Brown = high concentration; blue = low concentration. (**C**): Pathway mapping in naïve infected plants. (**D**): Pathway mapping in primed infected plants revealing some of the main metabolic pathways involved in the plants’ (naïve and primed) interaction with *Ptt*. The colour and sizes of the circles are based on *p* values and pathway impact values, respectively. The circles with large pathway impact values and small *p* values are significantly perturbed.

The metabolic reconfiguration of primed and naïve ‘Hessekwa’ barley plants following infection with *Ptt* at different time points involved primary metabolites such as amino acids, organic acids, fatty acids, and derivatives and secondary metabolites such as phenolic acids and derivatives, flavonoids, and alkaloids (Table S4). Phenolic acids and derivatives were the largest class of annotated discriminant metabolites, followed by flavonoids and fatty acids (Figure 4A). In addition, differential distribution patterns were observed on the generated heatmap dendrogram, highlighting differences in the relative concentrations of the metabolites between the infected and the control samples and between the naïve and primed infected samples (Figure 4B). Annotated metabolites included oxo-proline, citric acid, linolenic acid derivative isomer I, linolenoylglycerol, hordatine A hexose isomer II, hydroxytryptamine, isoscoparin 7-O-[6″-sinapoyl]-glucoside, and lutonarin (an isoorientin glucoside/a glucoside of luteolin), all discriminant metabolites in the primed infected samples. Discriminant metabolites in the naïve infected condition included hydroxylinolenic acid, hydroxyjasmonate sulphate, caffeoyl shikimate derivative isomer I and II, gallic acid monohydrate, and 6-prenylnaringenin (Table S4). The up- or down-regulation of these metabolites were characteristics of the infection status of the primed and naïve plants at different time points (2, 4, and 6 d.p.i.). Annotated metabolites were investigated for pathway analysis to reveal their implication in protection or defence mechanisms.

The MetPA module of the MetaboAnalyst bioinformatics tool package (http://www.metaboanalyst.ca/ (accessed on 30 November 2022) was used to determine the key metabolic pathways characterising the primed and naïve defence responses. The MetPA analyses of both states (naïve infected and primed infected) with the KEGG identifiers revealed the involvement of similar pathways but with different levels of significance and impact (Figure 3B,C; Tables S5 and S6). These included phenylpropanoid biosynthesis; phenylalanine (Phe), tyrosine (Tyr), and tryptophan (Trp) metabolism (both individually and as a group); and isoquinoline alkaloid biosynthesis. The primed infected plants displayed metabolites from the TCA cycle with moderate impact and significance. The mentioned pathways are among the most prevalent in the plant kingdom. In addition to having implications on defence against biotic and abiotic stresses, these pathways are also connected to the modulation of physiological and biochemical processes [43,44]. The flagged metabolic pathways and temporal dynamics of selected defence-related discriminant barley metabolites are discussed below. In general, these patterns indicate differential reprogramming over time (either high or low accumulation at specific time points that reflect early, late, or oscillatory responses) [45]. These fluctuations may mirror the availability of precursor metabolites, the flux through induced metabolic pathways, and the extent of degradation or conversion of specific metabolites.

#### 3.3.1. Amino Acids

**Figure 5:**
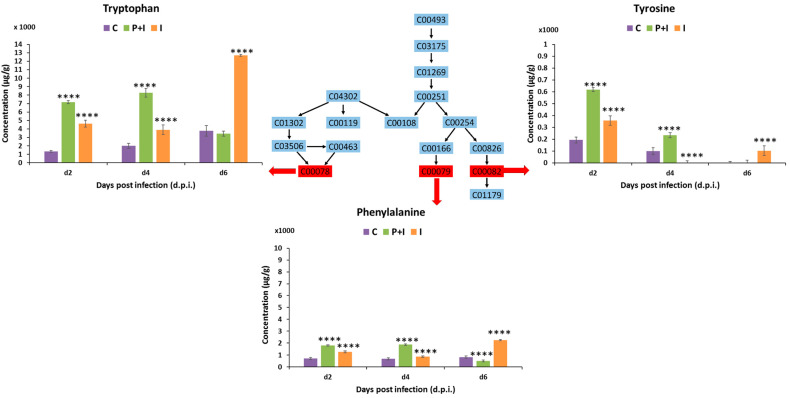
Illustration of the Phe, Tyr, and Trp biosynthesis pathways and MRM quantification of the main participants in the primed and naïve shoot tissues of barley infected with the fungal pathogen *P. teres* f. *teres*. The bar graphs were generated using the average integrated peak area (*n* = 9). The error bars represent the standard deviation. Concentrations are expressed in µg g^−1^ (Mean +/− standard deviation; Table S2). The statistical significance of the differences observed was evaluated using Student’s t-test, and all *p* values < 0.0001 (****) were considered as significant.

Significant pathways in both barley states also included the biosynthesis of Phe, Tyr, and Trp (Figure 5) and subsequent Phe and Trp metabolism. The MRM quantification profiles of the main components of the Phe, Tyr, and Trp biosynthesis pathways are shown via the red arrows in Figure 5 and Table S2. The concentrations of the three aromatic amino acids were significantly higher in the primed state at an early stage of infection (2 d.p.i. and 4 d.p.i). The concentration of these amino acids dropped at 6 d.p.i. in the primed plants and increased in the naïve infected plants. Alterations in the level of amino acids have previously been reported as a characteristic of the post-challenged phase [46,47]. Phe, Tyr, and Trp are mainly precursors in the biosynthesis of secondary metabolites, including all the ones mentioned above. In addition to their role as building blocks in protein synthesis, these amino acids are known for their actions in scavenging free radicals and the modulation of stomatal exposure and as osmolytes [48].

Other changes in amino acids included the up-regulation of oxo-proline (oxo-Pro) in the primed infected plants (Table S4). Plants accumulate Pro in response to several environmental stresses. Pro is a multi-functional amino acid, and its abundance may result from de novo synthesis or reduced degradation and utilisation. In the context of stress adaptation, Pro imparts stress tolerance by preserving the osmotic balance and cell turgidity and indirectly modulating the metabolism of ROS. In addition, the crosstalk of Pro with other osmoprotectants and signalling molecules like abscisic acid, glycine betaine, nitric oxide, hydrogen sulphide, etc., helps in strengthening the protective mechanism under stressful environments [49].

#### 3.3.2. Tricarboxylates and Fatty Acids

Specifically significant in primed infected plants, the TCA cycle is one of the key metabolic pathways. Changes affecting organic acids (e.g., through reversible transaminase reactions) and amino acids might affect nitrogen and carbon metabolism and link the associated metabolic cycles in support of anaplerotic reactions to replenish the citric acid cycle if it becomes depleted of intermediates in response to biosynthetic demands. Furthermore, alterations in carboxylic acid levels were reported in plants during stress responses, and it was suggested that the tricarboxylates could modulate signal transduction cascades linked to plant defence responses [50,51]. In this context, citrate and fumarate were reported as inducers of defence priming in *Arabidopsis thaliana* through complex signalling pathways [51].

Fatty acids (FAs) are ubiquitous, and their oxygenated derivatives (oxilipins) play crucial roles in the plant system [52]. Among the FAs annotated here, hydroxylinolenic and alpha-linolenic acids were found to be up-regulated in naïve infected barley at 2 and 4 d.p.i., respectively. In the same plants and at the early infection stage, 12-hydroxyjasmonate sulphate was also up-regulated. These compounds are precursors and/or active participants in signalling pathways and regulatory activities [53]. It has been suggested that FAs act by inserting themselves into the lipid bilayers of the fungal membrane. This results in the loss of fungal membrane integrity which uncontrollably releases proteins and electrolytes and eventually leads to the disintegration of the cell cytoplasm [54,55]. The antifungal activity of linolenic acid and other unsaturated FAs was assessed in a study conducted by Walters et al. [56], and the compound inhibits more than 50% of mycelial growth of a range of fungi, including *Pyrenophora avenae* and *Rhizoctonia solani*.

#### 3.3.3. Hydroxycinnamic Acids

**Figure 6:**
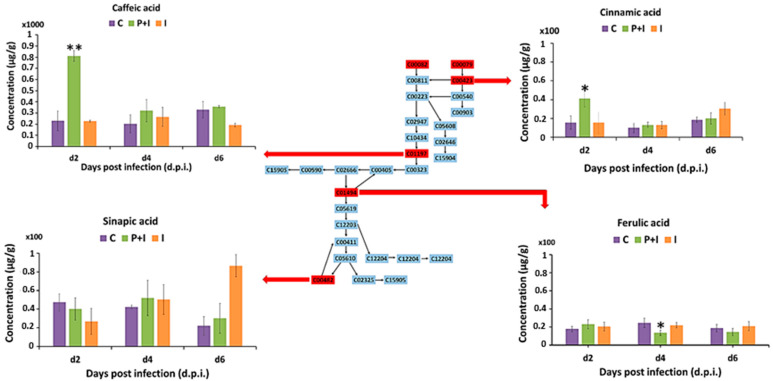
Illustration of the phenylpropanoid pathway and MRM quantification of main participants (cinnamic acid, caffeic acid, ferulic acid, and sinapic acid) in the primed and naïve shoot tissue of barley infected with the fungal pathogen *P. teres* f. *teres*. The bar graphs were generated using the average integrated peak area (*n* = 9). The error bars represent the standard deviation. Concentrations are expressed in µg g^−1^ (Mean +/− standard deviation; Table S2). The statistical significance of the observed differences was evaluated using Student’s *t*-test, and *p* values < 0.05 (*) and 0.01 (**) were considered as significant.

The most significant pathway in both the naïve and primed plants upon infection (as indicated by the topological characteristics) was the phenylpropanoid biosynthesis pathway, which results in the formation of cinnamic acid and hydroxylated derivatives such as caffeic acid, ferulic acid, and sinapic acid. The MRM quantification profiles of each phenolic acid are shown in Figure 6 and Table S2. Among all the phenolic acids, the concentration of cinnamic and caffeic acids significantly (*p* < 0.05) increased at the early infection stage (2 d.p.i.) in the primed infected plants compared to the control and the naïve infected plants. At 4 d.p.i., a significant decrease in ferulic acid was observed in the primed infected plants. These compounds might be associated with delayed infection or the faster response observed at 2 d.p.i. (Section 3.1). Interestingly, concentrations of sinapic acid were significantly higher in the naïve infected plant at 6 d.p.i. This suggests that sinapic acid (3,5-dimethoxy-4-hydroxycinnamic acid) is directly implicated in the barley–*P*. *teres* interaction and during the later stages of infection. Similarly to this study, in another study, rust infection in wheat crops was associated with an increase in ferulic and sinapic acid levels [57].

Phenolic acids are important specialised secondary metabolites of plants and are required for several physiological and mechanical processes [44,58]. Plants produce these phenolics for growth and development and, importantly, for protection (e.g., as phytoanticipin/phytoalexin antimicrobials and anti-oxidants, as well as for the strengthening of the cell wall through lignin synthesis and deposition). Through the activation of the phenylpropanoid pathway and associated sub-pathways, plants are shielded against upcoming attacks by phytopathogens and the spread of infection due to these induced defence mechanisms that are expressed both at the location of the attack and distant to it. As well as providing the chemical building blocks of the defence response, a network of interconnected signal transduction pathways regulates induced resistance, with phenolic acids serving as crucial signalling molecules [59,60].

#### 3.3.4. Hydroxycinnamic Acid Amides and Hordatines

**Figure 7:**
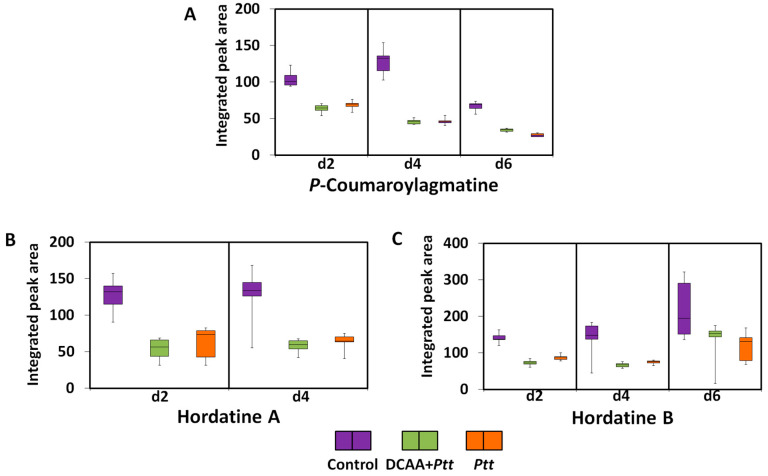
Box-and-whisker plots illustrating the relative quantitative profiles of discriminant barley-specific metabolites and a precursor resulting from priming treatment with 3,5-DCAA and infection with *Ptt*. (**A**): *p*-coumaroylagmatine, (**B**): hordatine A, and (**C**): hordatine B.

As phenolic acid derivatives, except for *p*-coumaroylhydroxyagmatine and *p*-coumaroylputrescine, all hydroxycinnamic acid amides (HCAAs) were down-regulated in both infected states and at all time points (Table S4). Backes et al. [41] reported the accumulation of coumaroylhydroxyagmatine in barley after infection with *Ptt*. Adding a hydroxyl group on coumaroylagmatine may increase the compound’s activity. Down-regulated HCAAs included the barley-specific metabolites hordatines A and B, as well as their precursor, *p*-coumaroylagmatine (Figure 7). The relative concentrations of these compounds were higher in the untreated control compared to infected plants (both naïve and primed), suggesting that their utilisation, conversion, or degradation is part of the defence against pathogen ingress.

Several studies have revealed the pivotal role of HCAAs in plant–pathogen interactions [61,62,63,64]. HCAA biosynthesis emanates from the phenylpropanoid pathway, and the occurrence in plants is associated with plant disease resistance. They confer cellular protection against pathogen attack by reinforcing cell walls or by acting directly as antimicrobial agents [61,65]. Hordatine A and B have been recognised as strong antifungal compounds of barley as early as in the 1960s [66,67,68,69]. The presence of these compounds as signatory metabolites reiterate their function in plant defence; however, the down-regulation observed might be associated with the level of susceptibility of the plant to *Ptt* and the inability to produce sustainable levels that allow for the plant to overcome the pathogen attack. It is also possible that the pathogen might target this important component of inducible plant defence as part of effector-triggered susceptibility (ETS). Phenolic compounds may protect against fungal attack through the inhibition of chitin and glycan biosynthesis, which results in the fungal plasma membrane and cell wall disruption. Such action causes the inhibition of metabolic enzymes and mitochondrial processes [44,59].

#### 3.3.5. Flavonoids

**Figure 8:**
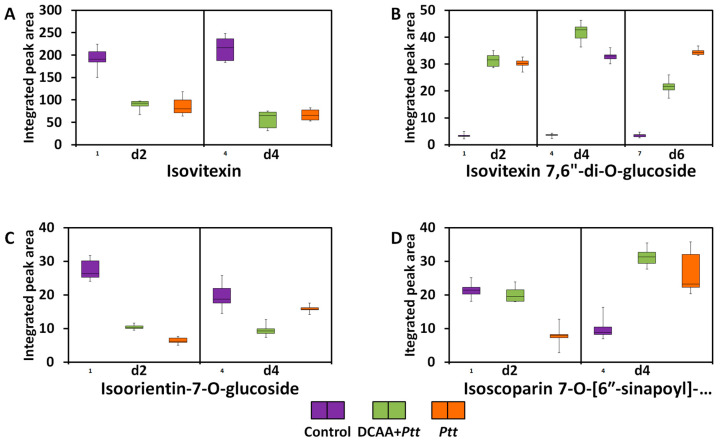
Relative quantitative profile of discriminant selected barley flavonoids (apigenin and luteolin derivatives) resulting from priming treatment with 3,5-DCAA and infection with *Ptt*. (**A**): isovitexin, (**B**): isovitexin 7,6″-di-O-glucoside, (**C**): isoorientin-7-O-glucoside (lutonarin), and (**D**): isoscoparin 7-O-[6″-sinapoyl]-glucoside.

Another metabolite class derived from the phenylpropanoid pathway is the flavonoid pathway. Among these, a prenylated naringenin was down-regulated in the naïve infected plants at a late stage (6 d.p.i.) (Table S4). Naringenin is a central precursor in the biosynthesis of flavonoids. Derivatives of apigenin and luteolin were also found to be discriminative across time points (Figure 8). The relative concentrations of isovitexin decrease at 2 and 4 d.p.i, while that of its diglycosylated derivative, isovitexin 7,6″-di-O-glucoside, increase. The relative concentrations of lutonarin were down-regulated at the early infection stage but higher in primed plants compared to the naïve ones. Similarly, at 2 d.p.i., isoscoparin 7-O-[6″-sinapoyl]-glucoside was down-regulated, and its concentration was higher in the primed infected plants than in the naïve infected plants. At 4 d.p.i., there was an up-regulation of the metabolite, and again, the level seemed higher in the primed infected plants than the naïve infected plants. Together with the phenolic acids, flavonoids are the most conserved class of compounds and the largest group of phytochemicals. They are involved in various processes such as auxin transport, signalling, plant growth, and pigmentation. The main function ascribed to flavonoids is their antioxidant abilities, which help to quench reactive oxygen species (ROS) in the host and the pathogen during plant response [70,71,72,73]. Lutonarin isolated from barley was demonstrated to have inhibitory activity on free radicals [74]. The capacity of flavonoids to inhibit the growth of fungal spores and limit the elongation of mycelium hyphae is well recognised [75,76]. It is believed that flavonoids contribute to strengthening plant structures and function as a physical barrier against fungus invasion [76]. In wheat and maize, flavonoids are involved in resistance against *F. graminearum* and *F. verticillioides*, respectively [77,78]; this study marks the first time in the literature that their role in plant defence against the fungal pathogen *P. teres* f. *teres* has been highlighted.

#### 3.3.6. Alkaloids

**Figure 9:**
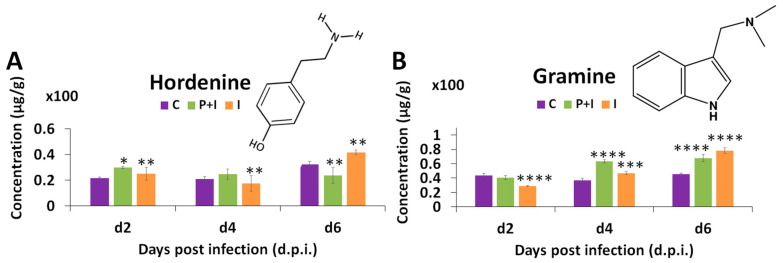
MRM quantification of hordenine and gramine from primed and naïve shoot tissues of barley infected with the fungal pathogen *P. teres* f. *teres*. The bar graphs were generated using the average integrated peak area (*n* = 9). The error bars represent the standard deviation. Concentrations are expressed in µg g^−1^ (Mean +/− standard deviation; Table S2). The statistical significance of the differences observed was evaluated using Student’s *t*-test, and *p* values < 0.05 (*), 0.01 (**), 0.001 (***), and 0.0001 (****) were considered as significant.

Furthermore, two important alkaloids in barley were also found and quantified using the MRM method as described in the Materials and Methods section: hordenine and gramine (Figure 9 and Table S2). The concentration of these metabolites fluctuated over time. However, hordenine concentration was significantly higher in the primed infected plants at 2 d.p.i. and in the naïve infected plants at 2 and 6 d.p.i. With gramine, higher concentrations were observed at 4 and 6 d.p.i. for all infected plant conditions. Hordenine, an isoquinoline alkaloid (second most impactful pathway; Figure 4A,B; Tables S5 and S6) and gramine, an indole alkaloid, were both reported as antifungal metabolites. Lovett and Hoult [79] demonstrated the inhibition capacity of both alkaloids against *Drechslera teres*, the anamorph stage of *Ptt.*

N-isoferuloylhydroxytryptamine and *p*-coumaroyltryptamine were also annotated as discriminant metabolites as they were up-regulated in both primed and naïve infected plants at 2 and 6 d.p.i. These are neutral HCAAs that are generally classified as alkaloids. Together with hydroxytryptamine (up-regulated at 2 d.p.i. in the primed infected plants), they were not previously identified as constituent metabolites in barley cultivar profiling [23,26]. However, in a recent study where DCAA and other dichlorinated compounds were used to induce systemic acquired resistance in barley, they were signatory biomarkers that accumulated in shoot tissues in response to treatment [22]. This points to their antifungal function in plant defence and their role as phytoalexins in barley. The antimicrobial activity of tryptamine derivatives was also described in barley under attack by *Fusarium culmorum*, responsible for Fusarium root rot disease [80], and the build-up of *p*-coumaroyltryptamine was noted in *A. thaliana* leaf tissue after 24 h of infection with *Pseudomonas syringae* [81].

### 3.4. Mapped Metabolic Networks

**Figure 10:**
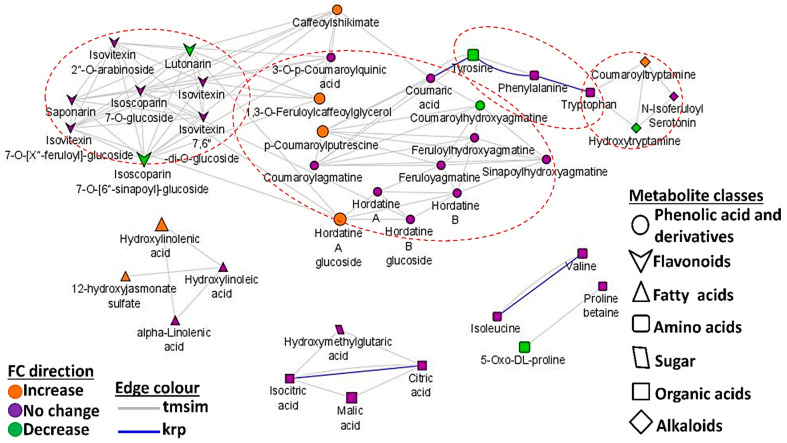
Mapped metabolic networks of all annotated metabolites identified via OPLS-DA analysis based on 2 d.p.i. data. The green nodes represent the metabolites that are up-regulated in the primed infected plants, and the orange ones represent those that are up-regulated in the naïve infected plants. There is an overlap between the class of phenolic acids and derivatives and that of flavonoids. The node size reflects the magnitude of the fold change (FC). The blue lines represent the connections of compounds via KEGG reaction pair (krp), and the grey lines connects the nodes (metabolites) based on their chemical similarity.

Metabolic (correlation) network analysis enables the characterisation of the complex relationship(s) in measured metabolites. Thus, in addition to the pathway enrichment analyses (Figure 4), a network demonstrating metabolic interaction patterns between metabolites identified by OPLS-DA (comparing the naïve and primed infected states) was constructed to understand the possible regulatory elements of the metabolic responses of primed and naïve plants to *Ptt* at the early infection stage (2 d.p.i.) (Figure 10). MetaMapp analysis makes use of statistical data such as fold changes and *p* values to construct metabolic networks showing significantly different metabolites in primed infected samples (green) and naïve infected plants (orange) [47]. Relational patterns in the experimental data are depicted by metabolic networks that identify altered graph neighbourhoods. Since these relationships are not dependent on predefined pathways, they allow for the description of the altered metabolomes established by new pathway connections as a result of the experimental conditions. For example, the agmatines and the hordatines are found in one cluster, indicating the structural relatedness, further affirming that the agmatines serve as precursors to the biosynthesis of the hordatines.

In addition, in the network topology, phenolic acids, flavonoids, and alkaloids, derived from caffeoylshikimate and amino acids such as Tyr, Phe, and Trp, appear to form the core hub. Isolated interaction could also be responsible for forming the fatty acid, organic acid, and other amino acid (proline betaine, oxo-proline, valine, and isoleucine) clusters. In addition, the biological interaction observed between Tyr, Phe, and Trp highlights the strong connection between these metabolites and reiterates their combined role as precursors of a broad spectrum of secondary metabolites. It can also be proposed that there is alignment between the production and utilisation of the three aromatic amino acids in regulating the production of these defensive secondary metabolites.

## 4. Conclusions

The mechanism(s) underlying plant priming is still very unclear at a biochemical level; however, two of the main characteristics are the early and long-lasting responses. This study focused on evaluating the dynamic intricacies of the metabolic changes in barley plants primarily preconditioned with 3,5-DCAA in response to a secondary stress—infection by *P. teres* f. *teres.* The primed plants were first characterised by a delay in the development of the distinctive visual symptoms associated with *P. teres* f. *teres* infection. A strong and rapid response was observed, portraying a state of alertness. However, this response was not maintained over time as the pathogen apparently managed to overcome the induced resistance.

These observations were further investigated using untargeted metabolomics (in combination with chemometric tools) and targeted metabolomic approaches which provided insights into the reconfigured primed and natural response of barley to *Ptt*. Metabolites and the measurement of dynamic changes in their profiles informatively reflected the differential and functional features of the naïve and 3,5-DCAA-treated plants’ metabolism in response to *Ptt* inoculation and subsequent infection. Both primary and secondary metabolism were affected, and one of the most significant pathways affected was the phenylpropanoid pathway. Higher concentrations of the aromatic amino acids Phe, Tyr, and Trp were found to be associated with the early responses in the barley-infected shoot tissues. Because the hordatine metabolites are well-known antifungal agents in barley plants, their down-regulation here may be linked to plant susceptibility due to the inability to synthesise sufficient amounts to repel the attack.

The variations observed at the beginning of infection (2 d.p.i.) in the primed plants were linked to the early and stronger response typical of the post-primed phase. The metabolites responsible for such delays and related to the priming response were highlighted; e.g., isovitexin-7,6″-diglucoside accumulated and was more prevalent in the primed plants at 2 and 4 d.p.i. Others included cinnamic and caffeic acids, citric acid, and oxo-proline. These findings support the speculation on the ability of 3,5-DCAA as a priming agent to enhance the defence response. Despite the aforementioned metabolic reconfiguration of the primed barley, the defence-related metabolite production therein was not sustainable to ward-off the attack. The secondary effects brought on by cell death may also impact and obfuscate the observable patterns in the data. The data obtained under the experimental conditions did not allow for the depiction of a possible mechanism underlying priming by 3,5-DCAA. However, the obtained descriptive insights serve as a departure point for further research on the use of dichlorinated inducers as priming agents in barley and other plants to manipulate/enhance the defence response to fungal attack and subsequent infection.

## Data Availability

The study design information, LC-MS data, data processing, and analyses are reported on and incorporated into the main text. Raw data, analyses and data processing information, and the meta-data were deposited into the Mass Spectrometry Interactive Virtual Environment, MassiVE (https://massive.ucsd.edu) at https://massive.ucsd.edu/MSV000092480/ (19 July 2023).

## Supplementary Materials

The following are available online at https://www.mdpi.com/article/10.3390/metabo13090997/s1: Figure S1. Disease triangle illustrating factors contributing to the progression of disease. Figure S2. Preliminary screening of cultivars from the Western Cape region of South Africa. Figure S3. Fungal growth before (A) and after (B) conidia induction under near-UV light (long wave). Figure S4. Ultra-high performance liquid chromatography—mass spectrometry (UHPLC–MS) base peak intensity (BPI) chromatograms (negative and positive ionisation) of barley treated with 3,5-DCAA and infected with *P. teres* f. *teres* and evaluated over 2, 4, and 6 d.p.i. Figure S5. Principal component analysis (PCA) score plots of ESI (+) data from shoot extracts of the ’Hessekwa’ cultivar of *Hordeum vulgare*. Table S1. Optimum parameters for UFLC–MRM–MS quantitative analysis listing targeted standards and standard curve equation for quantification. Table S2. Absolute quantification of selected metabolites in extracts from shoot tissues of primed and naïve barley plants following infection with *P. teres* f. *teres*. Table S3. Annotated metabolites used for correlation network analyses. Discriminant metabolites were extracted from OPLS-DA, comparing the naïve infected (reference) vs. primed infected metabolites (Condition_A). Table S4. List of all annotated (putatively identified) discriminant metabolites from leaves of the barley cultivar ‘Hessekwa’ (treated/untreated with 3,5-DCAA and infected with *P. teres* f. *teres* and harvested at 2, 4, and 6 d.p.i). Table S5. Metabolic pathways generated from Metabolomics Pathway Analysis (MetPA) in MetaboAnalyst 5.0 and involving annotated metabolites in primed infected barley plants. Table S6. Metabolic pathways generated from Metabolomics Pathway Analysis (MetPA) in MetaboAnalyst 5.0 and involving annotated metabolites in naïve infected barley plants.
