# Supplementary material for: Metabolomic Reconfiguration in Primed Barley (Hordeum vulgare) Plants in Response to Pyrenophora teres f. teres Infection
Source: Metabolites. 2023 Sep 7;13(9):997. doi: 10.3390/metabo13090997 (PMC10537252; doi:10.3390/metabo13090997)
Supplement: Supplementary file 1 [file metabolites-13-00997-s001.zip › metabolites-2575714-supplementary.pdf]

Supplementary File

Metabolomic reconfiguration in primed barley (*Hordeum vulgare* L.) plants in response to *Pyrenophora teres* f. *teres* infection

Claude Y. Hamany Djande, Fidele Tugizimana, Paul A. Steenkamp, Lizelle A. Piater and Ian A. Dubery*.

Research Centre for Plant Metabolomics, Department of Biochemistry, University of Johannesburg, P.O. Box 524, Auckland Park, Johannesburg 2006, South Africa; claudeh@uj.ac.za (C.Y.H.D.); ftugizimana@uj.ac.za (F.T.); psteenkamp@uj.ac.za (P.A.S.); lpiater@uj.ac.za (L.A.P.).

Research Centre for Plant Metabolomics, Department of Biochemistry, University of Johannesburg, P.O. Box 524, Auckland Park, Johannesburg 2006, South Africa; 201410297@student.uj.ac.za (C.Y.H.D.); psteenkamp@uj.ac.za (P.A.S.).

* Correspondence: idubery@uj.ac.za; Tel.: +27-11-5592401

Figure S1. Disease triangle illustrating factors contributing to the progression of disease.

Figure S2. Preliminary screening of cultivars from the Western Cape region of South Africa.

Figure S3. Fungal growth before (A) and after (B) conidia induction under near UV light (long wave).

Figure S4. Ultra–high performance liquid chromatography – mass spectrometry (UHPLC–MS) base peak intensity (BPI) chromatograms (negative and positive ionisation) of barley treated with 3,5-DCAA and infected with *P. teres* f. *teres* and evaluated over 2, 4 and 6 d.p.i.

Figure S5. Principal component analysis (PCA) score plots of ESI (–) and (+) data from shoot extracts of the 'Hessekwa' cultivar of *Hordeum vulgare*.

Table S1. Optimum parameters for the UFLC–MRM–MS quantitative analysis listing targeted standards and standard curve equation for quantification.

Table S2. Absolute quantification of selected metabolites in extracts from shoot tissues of primed and naïve barley plants following infection with *P. teres* f. *teres*.

Table S3. Annotated metabolites used for correlation network analyses. Discriminant metabolites were extracted from OPLS-DA comparing the naïve-infected (reference) *vs*. primed-infected metabolites (Condition_A).

Table S4. List of all annotated (putatively identified) discriminant metabolites from leaves of the barley cultivar 'Hessekwa' treated/untreated with 3,5-DCAA and infected with *P. teres* f. *teres* and harvested at 2, 4 and 6 d.p.i.

Table S5. Metabolic pathways generated from Metabolomics Pathway Analysis (MetPA) in MetaboAnalyst 5.0 and involving annotated metabolites in primed-infected barley plants.

Table S6. Metabolic pathways generated from Metabolomics Pathway Analysis (MetPA) in MetaboAnalyst 5.0 and involving annotated metabolites in naïve-infected barley plants.

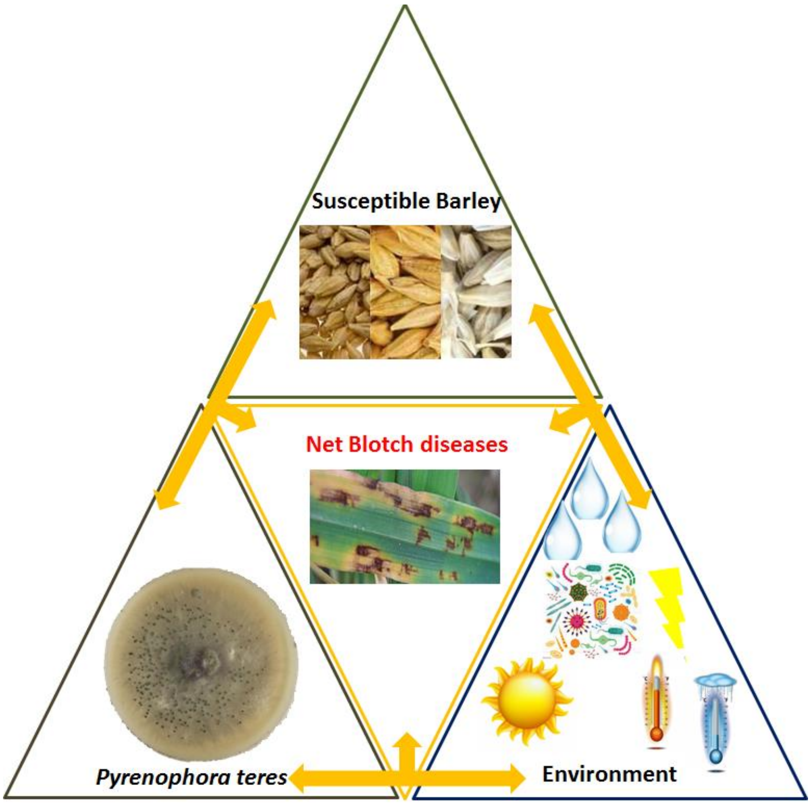

Figure S1. Disease triangle illustrating factors contributing to the progression of disease. The interaction of *P. teres* with a susceptible barley cultivar and in a favourable environment will result in net blotch diseases leading to important yield losses. The severity of the pathogen's spread relies heavily upon certain environmental factors, as the conidia require specific temperature (10-25 °C), relative humidity (95-100%), and leaf wetness for dispersal and germination.

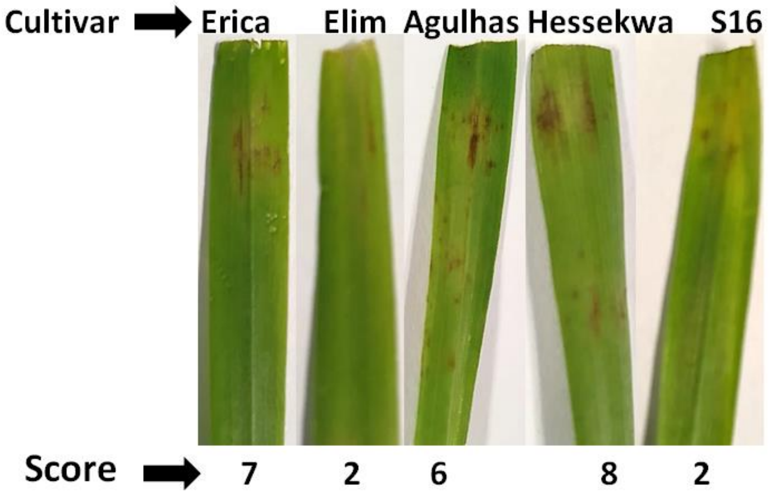

Figure S2. Preliminary screening of cultivars from the Western Cape region of South Africa. Barley shoot tissue segments were pressure infiltrated with a *Ptt* conidia suspension and the infection was monitered over 7 d.p.i. Net blotch net form (NBNF) disease symptoms were evaluated on a numerical scale ranging 0 to 10. 0=No symptoms; 1=Resistant; 2=Resistant to moderately resistant; 3=Moderately resistant; 4=Moderately resistant to moderately susceptible; 5=Moderately resistant to moderately susceptible; 6=Moderately resistant to moderately susceptible; 7=Moderately susceptible; 8=Moderately susceptible to susceptible; 9=Susceptible; 10=Very susceptible.

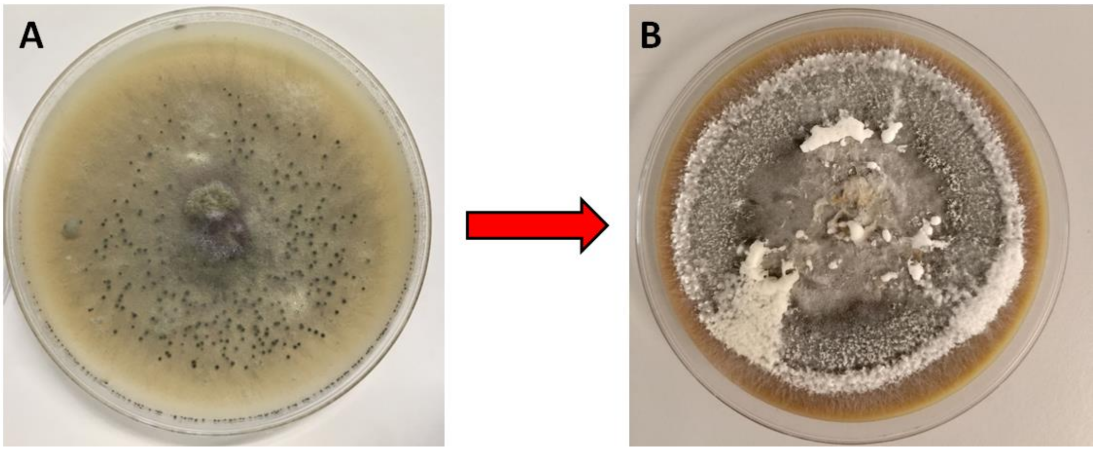

Figure S3. *Pyrenophora teres f. tere*s fungal growth before (**A**) and after (**B**) induction of conidia under near UV light (long wave). The fungus was first initiated on V8-PDA (V8 vegetable extract-potato dextrose agar) medium and sub-cultured into a barley-oat-agar (BOA, pH 7) solid media for improved sporulation The plates were incubated for 10 d under 12 h/12 h photoperiod at 22 °C. Ptt sporulation was induced by placing plates hydrated with 500 μL sterile water under black light (365 nmnear- UV light) for 20 h. To induce conidia formation, fungal plates were further incubated for 24 h at 15 °C in the dark (JM Gonzalez, personal communication).

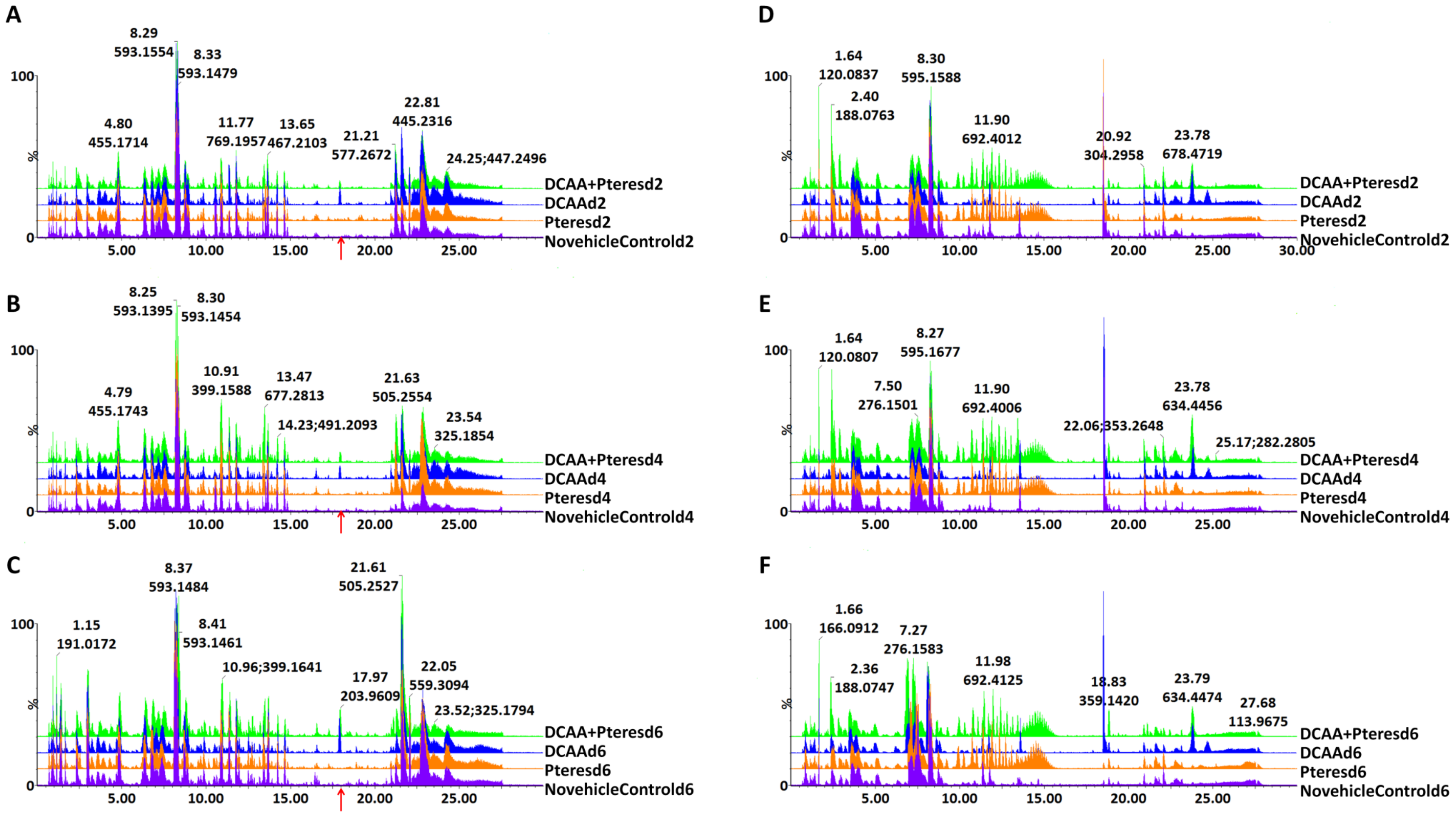

Figure S4. Ultra–high performance liquid chromatography – mass spectrometry (UHPLC–MS) base peak intensity (BPI) chromatograms (negative and positive ionisation) of barley treated with 3,5-DCAA and infected with *P. teres* f. *teres* and evaluated over 2, 4 and 6 d.p.i. **(A-C):** shoot extracts from 'Hessekwa', **ESI** negative data; **(D-F):** shoot extracts from 'Hessekwa', **ESI** positive data. The red arrows indicate the 3,5-DCAA ion peaks.

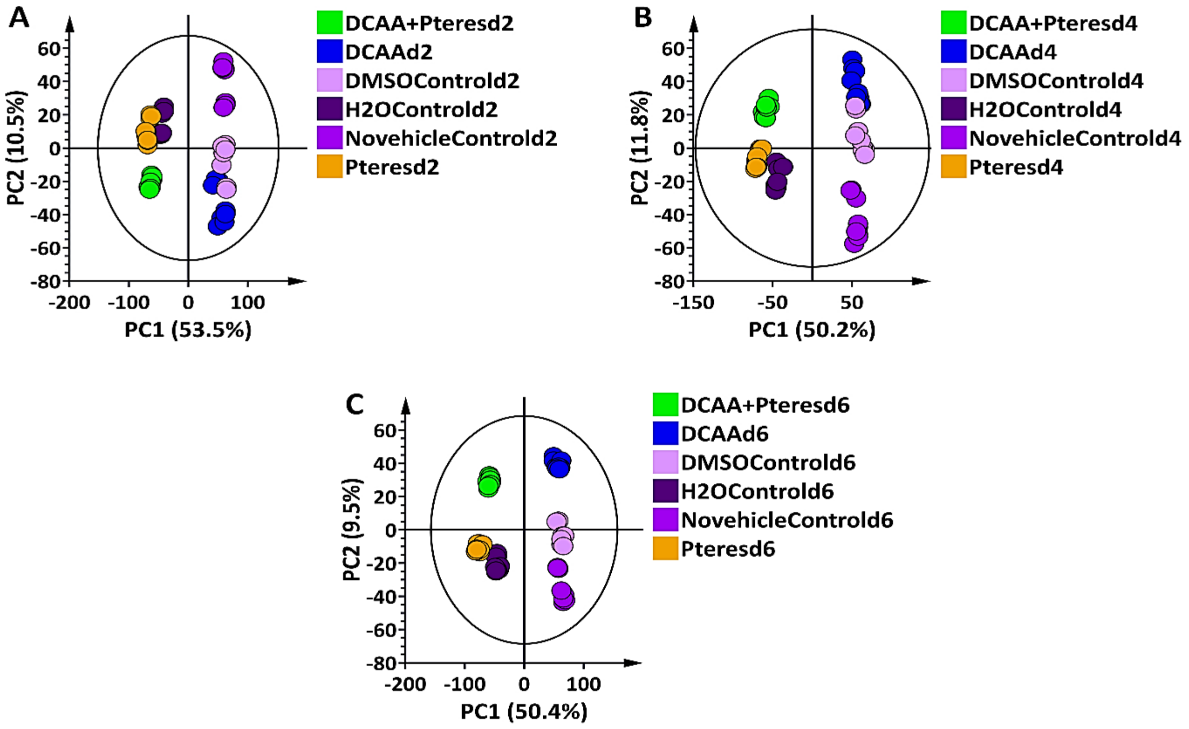

Figure S5. Principal component analysis (PCA) score plots of ESI (+) data from shoot extracts of the 'Hessekwa' cultivar of *Hordeum vulgare*. All data were *Pareto* scaled and the calculated Hotelling's T2 with a 95% confidence interval is represented by the ellipses present in each PCA score plot. (**A**): 7-component model of all conditions d2, explaining 83.6% variation and predicting 76.2% variation; (**B**): 5-component model of all conditions d4, explaining 78.3% variation and predicting 72.4% variation; (**C**): 6-component model of all conditions d2, explaining 83.6% variation and predicting 76.2% variation.

Table S1. Optimum parameters for the UFLC–MRM–MS quantitative analysis listing targeted standards and standard curve equation for quantification.

	Standards	Precursor ion	Rt (min)	CE	R^2	Equation
1	Tyrosine	182.0000>136.1000	3.77	-13.0	0.9833	$y=1x10^7x + 1x10^7$
2	Phenylalanine	166.0000>120.1000	4.69	-14.0	0.9964	$y=1x10^8x + 1x10^6$
3	Tryptophan	205.2000>188.0500>146.1000	8.12	-15.0<br>-15.0	0.9852	$y=1x10^7x + 1x10^7$
4	Cinnamic acid	149.1678>149.1678	14.58	-11.0	0.9856	y=308342x+ 04994
5	Ferulic acid	195.1878>195.1878	12.41	-8.0	0.9938	$y=5x10^6x + 37181$
6	Sinapic acid	225.2178>225.2178	12.88	-6.0	0.9986	$y=6x10^6x + 78870$
7	Caffeic acid	181.1678>181.1678	9.745	-8.0	0.9840	y=138945x + 58079
8	Gramine	175.2478>175.2478	12.28	-9.0	0.9928	$y=4x10^7x + 739066$
10	Hordenine	166.2378>166.2378	4.87	-10.0	0.9912	$y=1x10^8x + 3x10^6$

CE = collision energy (eV), R =regression coefficient, > = transition

Table S2. Absolute quantification of selected metabolites in extracts from shoot tissues of primed and naïve barley plants following infection with *P. teres* f. *teres*.

Metabolites	Concentration (μg.g^{-1} tissue)								
	2 d.p.i.			4 d.p.i.			6 d.p.i.		
	Control	Infected	Primed infected	Control	Infected	Primed infected	Control	Infected	Primed infected
**Amino acids**									
Phenylalanine	711.67±96	1272.42±57	1799.40±87	689.19±92	866.12±73	1877.25±68	836.73±88	2252.90±76	503.75±60
Tyrosine	192.9172±24	619.34±40	357.87±18	99.45626±28	233.98±16	1.39±22	0.00±0	102.47±16	0.00±0
Tryptophan	1347.93±111	4627.69±400	7152.21±187	2008.71±284	3895.35±521	8266.72±564	3787.65±631	12694.25±279	3444.06±95
**Phenolic acids**									
Cinnamic acid	15.61±7	15.67±9	41.04±11	10.14±5	13.03±5	13.20±4	18.63±3	30.40±7	20.09±6
Caffeic acid	228.96±88	226.32±74	811.77±81	202.57±90	263.86±82	320.44±14	330.37±49	190.58±99	356.92±11
Ferulic acid	17.81±3	20.43±5	23.21±4	24.60±5	21.95±4	13.53±3	18.74±5	21.09±5	14.47±3
Sinapic acid	47.40±9	26.78±14	40.24±12	42.33±02	50.38±16	52.12±19	22.03±10	86.45±12	30.22±16
**Alkaloids**									
Hordenine	21.59±1	25.12±2	29.88±2	21.00±1	17.57±6	24.65±4	32.40±5	41.66±6	23.59±4
Gramine	43.69±8	28.79±5	40.68±7	36.92±5	47.04±6	63.55±5	45.34±7	78.46±8	67.90±6

Quantitative values are expressed as μg/g tissue. The ± indicates the standard deviation of n = 9 determinations.

Table S3. Annotated metabolites used for correlation network analyses. Discriminant metabolites were extracted from OPLS-DA comparing the naïve-infected (reference) *vs.* primed-infected metabolites (Condition_A).

PubChem ID	KEGG ID	Simplified Molecular Input Line Entry System (SMILES)	Compound Name	*P*-value	Fold change
5202	C00780	C1=CC2=C(C=C1O)C(=CN2)CCN	Hydroxy-tryptamine	0.003	1.223
5280691	C04498	C1=CC(=CC=C1C=CC(=O)NCCCCN=C(N)N)O	Coumaroy-lagmatine	0.313	2.786
6305	C00078	C1=CC=C2C(=C1)C(=CN2)CC(C(=O)O)N	Tryptophan	0.330	1.030
6140	C00079	C1=CC=C(C=C1)CC(C(=O)O)N	Phenylalanine	0.386	1.579
6057	C00082	C1=CC(=CC=C1CC(C(=O)O)N)O	Tyrosine	0.000	1.659
525	C00149	C(C(C(=O)O)O)C(=O)O	Malic acid	0.001	0.609
311	C00158	C(C(=O)O)C(CC(=O)O)(C(=O)O)O	Citric acid	0.213	1.112
6287	C00183	CC(C)C(C(=O)O)N	Valine	0.018	0.822
1198	C00311	C(C(C(C(=O)O)O)C(=O)O)C(=O)O	Isocitric acid	0.739	0.982
6306	C00407	CCC(C)C(C(=O)O)N	Isoleucine	0.114	0.687
637542	C00811	C1=CC(=CC=C1C=CC(=O)O)O	Coumaric acid	0.117	0.926
162350	C01714	C1=CC(=CC=C1C2=CC(=O)C3=C(O2)C=C(C(=C3O)C4C(C(C(C(O4)CO)O)O)O)O)O	Isovitexin	0.757	0.905
442611	C05990	COC1=C(C=CC(=C1)C2=CC(=O)C3=C(O2)C=C(C(=C3O)C4C(C(C(C(O4)CO)O)O)O)O)O	Isoscoparin	0.019	3.818
44258179	C05990	COC1=C(C=CC(=C1)C2=CC(=O)C3=C(C(=C(C=C3O2)OC4C(C(C(C(O4)CO)O)O)O)C5C(C(C(C(O 5)CO)O)O)O)O)O	Isoscoparin 7-O-glucoside	0.617	1.031
5280934	C06427	CCC=CCC=CCC=CCCCCCCCC(=O)O	alpha-Linolenic acid	0.263	0.455
16061067	C07354	CCC=CCC(C(CC=CCCCCCCCC(=O)O)O)O	9K,12,13-diHODE	0.025	0.690
441381	C08064	C1=CC(=CC=C1C2=CC(=O)C3=C(C(=C(C=C3O2)OC4C(C(C(C(O4)CO)O)O)O)C5C(C(C(C(O5)C O)O)O)O)O)O	Saponarin	0.115	0.644
45485025	C08307	C1=CC(=CC=C1C2C(C3=C(O2)C=CC(=C3)C=CC(=O)NCCCCN=C(N)N)C(=O)NCCCCN=C(N)N) O	Hordatine A	0.460	2.110
72193633	C08308	COC1=CC(=CC2=C1OC(C2C(=O)NCCCCN=C(N)N)C3=CC=C(C=C3)O)C=CC(=O)NCCCCN=C(N)N	Hordatine B	0.478	2.040
7016562	C10172	C[N+]1(CCCC1C(=O)[O-])C	Proline betaine	0.286	0.678
5281762	C10434	C1C(C(C(C=C1C(=O)O)O)O)OC(=O)C=CC2=CC(=C(C=C2)O)O	Caffeoylshikimate	0.004	0.841

90478782	C12208	C1C(C(C(CC1(C(=O)O)O)OC(=O)C=CC2=CC=C(C=C2)O)O)O	3-O-p-Coumaroyl-quinic acid	0.093	0.934
10708957	C16346	CC(C=CCC=CCC=CCCCCCCCC(=O)O)O	Hydroxylinolenic acid	0.000	0.313
6439562	C18326	C1=CC(=CC=C1C=CC(=O)NCCCCN)O	p-Coumaroyl-putrescine	0.000	0.516
129892683		COC1=C(C=CC(=C1)C=CC(=O)C(C(CO)O)(C(=O)C=CC2=CC(=C(C=C2)O)O)O)O	1,3-O-Feruloyl-caffeoyl-glycerol	0.018	0.585
44257758		C1=CC(=CC=C1C2=CC(=O)C3=C(C(=C(C=C3O2)OC4C(C(C(C(O4)CO)O)O)O)C5C(C(C(C(O5)CO)O)O)OC6C(C(C(C(O6)CO)O)O)O)O)O	Isovitexin 7,6" -di-O-glucoside	0.149	1.694
44468060		C1C(C(C(C(O1)OC2C(C(C(OC2C3=C(C4=C(C=C3O)OC(=CC4=O)C5=CC=C(C=C5)O)O)CO)O)O)O)O)O	Isovitexin 2″-O-arabinoside	0.274	3.102
44559810		C1=CC(=C(C=C1C2=CC(=O)C3=C(C(=C(C=C3O2)OC4C(C(C(C(O4)CO)O)O)O)C5C(C(C(C(O5)CO)O)O)O)O)O)O)O	Lutonarin	0.000	1.651
44815853		C1CC(=O)C(C1CC(=O)O)CC=CCCOS(=O)(=O)O	12-hydroxy-jasmonate sulfate	0.006	0.886
46173376		COC1=C(C=CC(=C1)C=CC(=O)NCCCCN=C(N)N)O	Feruloyagmatine	0.189	0.650
71728415		COC1=C(C=C(C=C1)C=CC(=O)OCC2C(C(C(C(O2)OC3=C(C(=C4C(=C3)OC(=CC4=O)C5=CC=C(C=C5)O)O)C6C(C(C(C(O6)CO)O)O)O)O)O)O)O)O	Isovitexin 7-O-[X″-feruloyl]-glucoside	0.084	0.925
75536015		CCCC(CC=CCC=CCCCCCCCC(=O)O)O	Hydroxylinoleic acid	0.087	0.707
129830108		COC1=CC(=CC(=C1O)OC)C=CC(=O)N(CCCCN=C(N)N)O	Sinapoylhydroxy-agmatine	0.514	0.834
129852215		COC1=C(C=CC(=C1)C=CC(=O)N(CCCCN=C(N)N)O)O	Feruloylhydroxy-agmatine	0.877	0.893
131751022		COC1=CC(=CC2=C1OC(C2C(=O)NCCCCN=C(N)N)C3=CC=C(C=C3)OC4C(C(C(C(O4)CO)O)O)O)C=CC(=O)NCCCCN=C(N)N	Hordatine B glucoside	0.501	1.971
131751024		C1=CC(=CC=C1C2C(C3=C(O2)C(=CC(=C3)C=CC(=O)NCCCCN=C(N)N)O)C(=O)NCCCCN=C(N)N)OC4C(C(C(C(O4)CO)O)O)O	Hordatine A glucoside	0.001	0.326
131698836		C1CC(=O)NC1C(=O)O	5-Oxo-proline	0.000	1.586
10329583		C(C(CC(=O)O)CO)C(=O)O	Hydroxymethyl-glutaric acid	0.213	0.748
129694157		C1=CC(=CC=C1C=CC(=O)N(CCCCN=C(N)N)O)O	Coumaroylhydroxy-agmatine	0.008	1.148
51351495		COC1=C(C=C(C=C1)C=CC(=O)NCCC2=CNC3=C2C=C(C=C3)O)O	N-Isoferuloyl-serotonin	0.287	1.325
5458878		C1=CC=C2C(=C1)C(=CN2)CCNC(=O)C=CC3=CC=C(C=C3)O	Coumaroyl-tryptamine	0.001	0.796

Table S4. List of all annotated (putatively identified) discriminant metabolites from shoots of the barley cultivar 'Hessekwa' treated/untreated with 3,5-DCAA and infected with *P. teres* f. *teres* and harvested at 2, 4 and 6 d.p.i. The features were extracted from the multivariate OPLS-DA S-plots and the fold changes were calculated using a SIMCA software algorithm and are indicated where a metabolite was selected as a significant biomarker, either up- or down-regulated.

	Ionisation mode	Compound names	Rt (min)	*m/z*	DCAA+*Ptt*			*Ptt*		
					2 d.p.i.	4 d.p.i.	6 d.p.i.	2 d.p.i.	4 d.p.1.	6 d.p.i.
		**Amino acids**								
1	Pos	Tyrosine*	1.13	182.082	Up	Up		Up	Up	Up
2	Neg	5-Oxo-DL-proline	1.14	128.033	Up		Up			
3	Neg	Phenylalanine*	1.63	164.069	Up	Up	Down	Up		
4	Neg	Tryptophan*	2.40	203.081	Up	Up		Up	Up	Up
		**Organic acids**								
5	Neg	Citric acid	1.14	191.018				Up		
		**Phenolic acids and derivatives**								
6	Neg	Caffeoylshikimate derivative isomer I	10.24	679.261				Up		Up
7	Neg	Caffeoylshikimate derivative isomer II	10.59	679.261						Up
8	Neg	Coumaroylglucosylglycerol	10.86	399.163		Up		Up	Up	Up
9	Neg	Gallic acid monohydrate	12.41	187.096						Up
10	Neg	1,3-O-Feruloylcaffeoylglycerol	12.78	429.175			Up			Up
11	Pos	Cinnamic acid*	14.58	149.167	Up					
12	Pos	Caffeic acid*	9.745	181.167	Up					
13	Pos	Ferulic acid*	12.41	195.187		Down				
14	Pos	Sinapic acid*	12.88	225.217				Down		Up
15	Neg	Coumaroylhydroxyagmatine	2.46	291.146	Up			Up		
16	Pos	Coumaroylagmatine	3.92	277.164		Down			Down	
17	Neg	p-Coumaroylputrescine	2.32	235.145				Up		
18	Neg	Sinapoylhydroxyagmatine	6.36	351.127	Down				Down	
19	Pos	Hordatine B glucoside	3.58	372.181	Down	Down	Down	Down	Down	Down
20	Pos	Hordatine A glucoside isomer I	3.87	357.177			Down			Down
21	Neg	Hordatine A glucoside isomer II	4.01	757.353	Down	Down				
22	Pos	Hordatine B	7.18	291.156	Down	Down	Down	Down	Down	Down

23	Pos	Hordatine A	7.56	276.151	Down	Down	Down	Down	Down	
		**Flavonoids**								
24	Pos	Lutonarin	6.38	611.159	Up					
25	Pos	Saponarin	8.18	595.167	Down	Down	Down	Down	Down	Down
26	Pos	Isovitexin 7-O-rhamnosylglucoside	8.67	741.22	Down	Down	Down	Down	Down	Down
27	Neg	Isoscoparin 7-O-glucoside	8.87	623.16	Down	Down		Down	Down	
28	Neg	Isoscoparin 7-O-[6″-sinapoyl]-glucoside	11.48	829.221	Up					
29	Neg	Isovitexin 7,6" -di-O-glucoside	10.43	755.301	Up			Up		Up
30	Neg	Isovitexin	10.53	431.097	Down	Down		Down	Down	
31	Neg	Isovitexin 7-O-[6″-sinapoyl]-glucoside	11.36	799.209		Up	Down	Down	Up	Down
32	Neg	Isovitexin 7-O-[X″-feruloyl]-glucoside	11.78	769.199	Down	Down	Down	Down	Down	Down
33	Neg	6-Prenylnaringenin	19.09	339.217						Down
		**Alkaloids**								
34	Pos	Hordenine*	4.87	166.237	Up		Down	Down	Down	Up
35	Pos	Gramine*	12.28	175.247		Up	Up	Down	Up	Up
36	Neg	N-Isoferuloyl serotonin	2.42	351.129			Up			Up
37	Neg	Coumaroyltryptamine	2.55	289.13	Up		Up	Up		Up
38	Pos	Hydroxytryptamine	1.64	177.104	Up					
		**Fatty acids and derivatives**								
39	Neg	12-hydroxyjasmonate sulphate	4.36	305.069				Up		
40	Neg	Linolenic derivative I	20.95	675.358	Up					
41	Neg	Hydroxylinolenic acid	21.57	293.211				Up		
42	Neg	alpha-Linolenic acid	22.05	277.216					Up	
43	Pos	Linolenoylglycerol	22.07	353.267			Down			
44	Neg	Linolenic derivative II	22.81	445.233	Up		Down		Up	Down

**(***) metabolites quantified using the MRM method.

Table S5. Metabolic pathways generated from Metabolomics Pathway Analysis (MetPA) in MetaboAnalyst 5.0 and involving annotated metabolites in **primed-infected** barley plants.

**Pathway Name**	***p*-value**	**Impact**
Phenylpropanoid biosynthesis	$6.59E^{-07}$	0.048
Phenylalanine, tyrosine and tryptophan biosynthesis	$1.35E^{-03}$	0.021
Tryptophan metabolism	0.002	0.249
Phenylalanine metabolism	0.007	0.423
Aminoacyl-tRNA biosynthesis	0.011	0
Ubiquinone and other terpenoid-quinone biosynthesis	0.052	0
Biosynthesis of secondary metabolites - unclassified	0.052	0
Isoquinoline alkaloid biosynthesis	0.063	0.411
Tropane, piperidine and pyridine alkaloid biosynthesis	0.083	0
Flavone and flavonol biosynthesis	0.122	0
Tyrosine metabolism	0.177	0.167
Citrate cycle (TCA cycle)	0.195	0.115
Biosynthesis of unsaturated fatty acids	0.212	0
Starch and sucrose metabolism	0.212	0.089
Glutathione metabolism	0.254	0.012
Galactose metabolism	0.254	0.042
alpha-Linolenic acid metabolism	0.254	0.114
Arginine and proline metabolism	0.262	0.003
Glyoxylate and dicarboxylate metabolism	0.270	0.007
Glycine, serine and threonine metabolism	0.302	0

Table S6. Metabolic pathways generated from Metabolomics Pathway Analysis (MetPA) in MetaboAnalyst 5.0 and involving annotated metabolites in **naïve-infected** barley plants.

**Pathway Name**	***p*-value**	**Impact**
Phenylalanine, tyrosine and tryptophan biosynthesis	$3.75E^{-04}$	0.022
Tryptophan metabolism	$4.29E^{-04}$	0.241
Phenylpropanoid biosynthesis	0.002	0.051
Aminoacyl-tRNA biosynthesis	0.003	0
Isoquinoline alkaloid biosynthesis	0.042	0.411
Tropane, piperidine and pyridine alkaloid biosynthesis	0.056	0
Stilbenoid, diarylheptanoid and gingerol biosynthesis	0.076	0.115
Flavone and flavonol biosynthesis	0.082	0
Phenylalanine metabolism	0.082	0.423
Tyrosine metabolism	0.122	0.168
Biosynthesis of unsaturated fatty acids	0.147	0
Starch and sucrose metabolism	0.147	0.089
Galactose metabolism	0.177	0.042
alpha-Linolenic acid metabolism	0.177	0.114
Arginine and proline metabolism	0.183	0.003
Glycine, serine and threonine metabolism	0.212	0
Ubiquinone and other terpenoid-quinone biosynthesis	0.224	0
Flavonoid biosynthesis	0.290	0.021
